# Supplementary material for: Three New Species of Microdochium (Sordariomycetes, Amphisphaeriales) on Miscanthus sinensis and Phragmites australis from Hainan, China
Source: J Fungi (Basel). 2022 May 27;8(6):577. doi: 10.3390/jof8060577 (PMC9224723; doi:10.3390/jof8060577)
Supplement: Supplementary file 1 [file jof-08-00577-s001.zip › Table S1.pdf]

Table S1. Specimens and GenBank accession numbers of DNA sequences used in this study

Species	Voucher	Host /Substrate	Country	GeneBank accession numbers			
				LSU	ITS	BTUB	RPB2
Idriella lunata	CBS 204.56*	*Fragaria chiloensis*	USA	KP858981	KP859044	–	–
Microdochium albescens	CBS 291.79	*Oryza sativa*	Ivory Coast	KP858932	KP858996	KP859059	KP859105
	CBS 243.83	*Oryza sativa*	Unknown country	KP858930	KP858994	KP859057	KP859103
M. bolleyi	CBS 540.92	*Hordeum vulgare*	Syria	KP858946	KP859010	KP859073	KP859119
*M. chrysanthemoide*s	CGMCC3.17929*= LC5363	unnamed Karst Cave	China	KU746736	KU746690	–	–
	CGMCC3.17930* = LC5466	unnamed Karst Cave	China	KU746735	KU746689	–	–
M. citrinidiscum	CBS 109067*	*Eichhornia crassipes*	Peru	KP858939	KP859003	KP859066	KP859112
M. colombiense	CBS 624.94*	*Musa sapientum*	Colombia	KP858935	KP858999	KP859062	KP859108
M. dawsoniorum	BRIP 65649	*Sporobolus*	Australia	–	MK966337	–	–
M. fisheri	CBS 242.90*	*Oryza sativa*	UK	KP858951	KP859015	KP859079	KP859124
M. hainanense	SAUCC210781*	*Phragmites australis*	China	OM959323	OM956295	OM981146	OM981153
	SAUCC210782	*Phragmites australis*	China	OM959324	OM956296	OM981147	OM981154
M. indocalami	SAUCC1016*	*Indocalamus longiauritus*	China	MT199878	MT199884	MT435653	MT510550
M. lycopodinum	CBS 146.68	Air sample	The Netherlands	KP858929	KP858993	KP859056	KP859102
	CBS 109397	*Phragmites australis*	Germany	KP858940	KP859004	KP859067	KP859113
	CBS 109398	*Phragmites australis*	Germany	KP858941	KP859005	KP859068	KP859114
M. majus	CBS 741.79	*Triticum aestivum*	Germany	KP858937	KP859001	KP859064	KP859110
M. miscanthi	SAUCC211092*	*Miscanthus sinensis*	China	OM957532	OM956214	OM981141	OM981148
	SAUCC211093	*Miscanthus sinensis*	China	OM957533	OM956215	OM981142	OM981149

	SAUCC211094	*Miscanthus sinensis*	China	OM957534	OM956216	OM981143	OM981150
M. musae	CBS 111018 = CPC 5380	*Musa* cv. *Cavendish*	Costa Rica	–	AY293061	–	–
	CBS 143499 = CPC 32809	*Musa* sp	Malaysia	MH107941	MH107894	–	–
	CBS 143500* = CPC 32689	*Musa* sp	Malaysia	MH107942	MH107895	–	MH108003
	CPC 11234	*Musa* sp	Mauritius	MH107943	MH107896	–	–
	CPC 11240	*Musa* sp	Mauritius	MH107944	MH107897	–	–
	CPC 16258	*Musa* sp	Mexico	MH107945	MH107898	–	–
	CPC 32681	*Musa* sp	Malaysia	MH107946	MH107899	–	–
M. neoqueenslandicum	CBS 445.95	*Juncus effusus*	The Netherlands	KP858933	KP858997	KP859060	KP859106
	CBS 108926*	*Agrostis* sp	New Zealand	KP858938	KP859002	KP859065	KP859111
M. nivale	CBS 116205*	*Triticum aestivum*	UK	KP858944	KP859008	KP859071	KP859117
M. nivale var. *nivale*	CBS 288.50	Unknown	Unknown country	MH868135	MH856626	–	–
M. novae-zelandiae	CBS 143847	From turf leaves (Poaceae)	New Zealand	–	LT990655	LT990608	LT990641
	CPC 29693	From turf leaves (Poaceae)	New Zealand	–	LT990656	LT990609	LT990642
M. paspali	HK-ML-1371	*Paspalum vaginatum*	China	–	KJ569509	KJ569514	–
	QH-BA-48	*Paspalum vaginatum*	China	–	KJ569510	KJ569515	–
	SY-LQG66	*Paspalum vaginatum*	China	–	KJ569511	KJ569516	–
	WC-WC-85	*Paspalum vaginatum*	China	–	KJ569512	KJ569517	–
	WN-BD-452	*Paspalum vaginatum*	China	–	KJ569513	KJ569518	–
M. phragmitis	CBS 285.71*	*Phragmites australis*	Poland	KP858949	KP859013	KP859077	KP859122
	CBS 423.78	*Phragmites communis*	Germany	KP858948	KP859012	KP859076	KP859121
M. ratticaudae	BRIP 68298	introduced giant rat’s tail grasses	Australia	MW481666	MW481661	–	MW626890

M. rhopalostylidis	CPC 34449 = CBS 145125*	*Rhopalostylis sapida*	New Zealand	MK442532	MK442592	–	MK442667
M. seminicola	KAS3576 = CBS 139951 *	Maize kernels	Switzerland	KP858974	KP859038	KP859101	KP859147
	KAS1516 = CPC 26001	grain	Canada	KP858961	KP859025	KP859088	KP859134
	KAS3574 = DAOM 250155	Maize kernels	Switzerland	KP858973	KP859037	KP859100	KP859146
	KAS3158 = DAOM 250161	*Triticum aestivum*	Canada	KP858970	KP859034	KP859097	KP859143
	KAS1527 = DAOM 250165	grain	Canada	KP858966	KP859030	KP859093	KP859139
	KAS1473 = DAOM 250176	*Triticum aestivum*	Canada	KP858955	KP859019	KP859082	KP859128
M. sinense	SAUCC211097*	*Miscanthus sinensis*	China	OM959225	OM956289	OM981144	OM981151
	SAUCC211098	*Miscanthus sinensis*	China	OM959226	OM956290	OM981145	OM981152
M. sorghi	CBS 691.96	*Sorghum halepense*	Cuba	KP858936	KP859000	KP859063	KP859109
Microdochium sp.	SAUCC1017	*Indocalamus longiauritus*	China	MT199879	MT199885	MT435654	–
M. tainanense	CBS 269.76*	*Saccharum officinarum*	Taiwan	KP858945	KP859009	KP859072	KP859118
	CBS 270.76	*Saccharum officinarum*	Taiwan	KP858931	KP858995	KP859058	KP859104
M. trichocladiopsis	CBS 623.77*	*Triticum aestivum*	Unknown country	KP858934	KP858998	KP859061	KP859107
M. yunnanense	SAUCC1011*	*Indocalamus longiauritus*	China	MT199875	MT199881	MT435650	MT510547
	SAUCC1012	*Indocalamus longiauritus*	China	MT199876	MT199882	–	MT510548
	SAUCC1015	*Indocalamus longiauritus*	Chima	MT199877	MT199883	MT435652	MT510549
	SAUCC1018	*Indocalamus longiauritus*	Chima	MT199880	MT199886	MT435655	–

Isolates marked with "*" are ex-type, ex-epitype or ex-holotype strains.
